# Adipose Tissue Protects against Hepatic Steatosis in Male Rats Fed a High-Fat Diet plus Liquid Fructose: Sex-Related Differences

**DOI:** 10.3390/nu15183909

**Published:** 2023-09-08

**Authors:** Roger Bentanachs, Laia Blanco, Maria Montesinos, Aleix Sala-Vila, Iolanda Lázaro, Jose Rodríguez-Morató, Rosa María Sánchez, Juan Carlos Laguna, Núria Roglans, Marta Alegret

**Affiliations:** 1 Department of Pharmacology, Toxicology and Therapeutic Chemistry, School of Pharmacy and Food Science, University of Barcelona, 08028 Barcelona, Spain; bentanachs@ub.edu (R.B.); laiablanco.1999@gmail.com (L.B.); mariamontesinosg7@gmail.com (M.M.); rmsanchez@ub.edu (R.M.S.); jclagunae@ub.edu (J.C.L.); 2 Institute of Biomedicine, University of Barcelona, 08028 Barcelona, Spain; 3 IMIM-Hospital del Mar Medical Research Institute, 08003 Barcelona, Spain; asala3@imim.es (A.S.-V.); ilazaro@imim.es (I.L.); 4 Spanish Biomedical Research Centre in Physiopathology of Obesity and Nutrition (CIBEROBN), Instituto de Salud Carlos III (ISCIII), 28029 Madrid, Spain; jose.rodriguez@upf.edu; 5 Department of Medicine and Life Sciences, Universitat Pompeu Fabra, 08003 Barcelona, Spain

**Keywords:** adipose tissue, fructose, high-fat diet, leptin, non-esterified fatty acids

## Abstract

Non-alcoholic fatty liver disease is a sexual dimorphic disease, with adipose tissue playing an essential role. Our previous work showed that female rats fed a high-fat high-fructose diet devoid of cholesterol (HFHFr) developed simple hepatic steatosis dissociated from obesity. This study assessed the impact of the HFHFr diet on the male rat metabolism compared with data obtained for female rats. A total of 16 Sprague Dawley (SD) male rats were fed either a control (standard rodent chow and water) or HFHFr (high-fat diet devoid of cholesterol, plus 10% fructose in drinking water) diet for 3 months. Unlike female rats, and despite similar increases in energy consumption, HFHFr males showed increased adiposity and hyperleptinemia. The expression of hormone-sensitive lipase in the subcutaneous white adipose tissue was enhanced, leading to high free fatty acid and glycerol serum levels. HFHFr males presented hypertriglyceridemia, but not hepatic steatosis, partially due to enhanced liver PPARα-related fatty acid β-oxidation and the VLDL-promoting effect of leptin. In conclusion, the SD rats showed a sex-related dimorphic response to the HFHFr diet. Contrary to previous results for HFHFr female rats, the male rats were able to expand the adipose tissue, increase fatty acid catabolism, or export it as VLDL, avoiding liver lipid deposition.

## 1. Introduction

The influence of sex on the establishment of non-alcoholic fatty liver disease (NAFLD) is still a matter of debate. Although most general population studies indicate a higher prevalence of NAFLD in men than in women [1], there are also data suggesting that the prevalence of NAFLD is higher in female than in male adolescents [2]. Moreover, the incidence and prevalence of NAFLD increase in women after menopause, with hormone replacement therapy in post-menopausal women lowering the prevalence of NAFLD, thus indicating a protective effect of estrogen [3]. Estrogens play a key role in the regulation of lipid metabolism, which may explain, in part, the sex-related differences in the susceptibility to hepatic steatosis. Thus, it has been shown that estradiol suppresses hepatic *de novo* lipogenesis and cholesterol synthesis, while increasing fatty acid oxidation and the export of liver triglycerides via VLDL [4]. Moreover, estrogens control white adipose tissue distribution and its health by blocking hypertrophy and fibrosis and promoting vascularization [5]. Not only hormones, but also ethnicity and differences in risk factors, such as obesity and insulin resistance, could differently affect the development of NAFLD in each sex. For example, in a study examining hepatic triglyceride contents in 2287 subjects from a population-based sample, among Caucasians, the prevalence of hepatic steatosis was greater in men than in women, but this was not the case in African Americans or Hispanics [6]. Meanwhile, a meta-analysis comparing the characteristics of lean and overweight/obese patients with NAFLD showed that the percentage of females with lean NAFLD was higher [7], although another study found no influence of sex [8].

The use of animal models may help unravel the influence of sex on NAFLD establishment and development, avoiding the social and environmental gender influence which is present in human studies. Most studies on NAFLD are still being performed using male animal models by mirroring non-alcoholic steatohepatitis (NASH) or NASH associated with hepatocellular carcinoma. Our research group has been investigating fructose-associated metabolic alterations in female animal models for decades. Recently, we characterized a model of simple hepatic steatosis in female rats by administering a plant-based high-fat diet, rich in palmitic and stearic acids and devoid of cholesterol, that was supplemented with 10% fructose in the drinking water (high-fat high-fructose, HFHFr). Interestingly, in the female rats fed the HFHFr diet, hepatic steatosis was dissociated from obesity, as the white adipose tissue weight was not altered, despite the significant increase in calorie consumption when compared to the chow-fed rats [9].

Considering possible sex differences in the response to the HFHFr diet, we repeated the experimental protocol of the previous study, which consisted of administering either chow or the HFHFr diet for 3 months, using male rats. Here, we present data showing that the response to the HFHFr diet was different, depending on the sex of the animals. In contrast to our previous findings in female rats [9], the HFHFr diet did not cause hepatic steatosis in males, as assessed by Oil Red O (ORO) staining of liver sections, but elicited strong hypertriglyceridemia and increased adiposity in all the fat depots.

## 2. Materials and Methods

### 2.1. Animals and Diets

Sixteen male SD rats, aged two months (Envigo, Barcelona, Spain), were housed two per cage and randomly assigned to two groups (*n* = 8 in each). The control group (CT) was fed regular chow *ad libitum* (2018 Teklad Global rodent diet, Envigo, Barcelona, Spain) and had free access to water. The diet HFHFr group was fed a high fat diet *ad libitum* (Teklad Custom Diet TD. 180 456, Envigo, Madison, WI, USA) and had free access to a 10% *w*/*v* fructose solution. The compositions of the CT and HFHFr diets were the same as those reported in our previous study in female rats [9], and are shown in the Supplementary Material (Table S1). All animals were kept under conditions of constant humidity (40–60%) and temperature (20–24 °C), with a 12 h light/12 h dark cycle. Fructose beverages were changed and controlled three times a week. Solid food consumption and body weight were measured once a week. All procedures were performed under the principles and procedures outlined in the guidelines established by the Bioethics Committee of the University of Barcelona (Autonomous Government of Catalonia Act 5/21 July 1995). The Animal Experimentation Ethics Committee of the University of Barcelona approved all the experimental procedures involving animals (approval no. 10 106). At the end of the treatment, the rats were fasted for 2 h, and blood, serum, and tissue samples were obtained, as described previously [9]. Samples of sWAT and pWAT from the control and HFHFr-fed female rats were obtained from our previous study [9].

### 2.2. Serum Analysis

Serum adiponectin, insulin, leptin, and FGF21 concentrations were determined using specific ELISA kits from Millipore (Billerica, MA, USA). The colorimetric kit to measure non-esterified fatty acids (NEFA) was obtained from FUJIFILM Wako Chemicals (Neuss, Germany), and the kits to assess alanine (ALT) and aspartate aminotransferase (AST) kinetics were acquired from Spinreact (Girona, Spain). The insulin sensitivity index (ISI) was calculated as

$$\frac{2}{\left[insulin\;(µM)\times blood\;glucose\;(µM)+1\right]}$$

### 2.3. Liver Lipid Content

Hepatic TAG and cholesterol were extracted as described by Qu et al. [10] and determined using colorimetric assay kits (41030 and MD41021, respectively) from Spinreact (Girona, Spain).

### 2.4. Liver Lipidomic Analysis

Levels of fatty acid methyl esters from TAG were determined by gas chromatography/electron ionization/mass spectrometry after TAG isolation by solid-phase extraction, and the levels of diacylglycerols (DAG) and ceramides (Cer), were determined by liquid chromatography-tandem mass spectrometry (LC–MS/MS), as described previously [11], with minor modifications. Briefly, six DAG species (namely, DAG 16:0/16:0, DAG 18:0/18:0, DAG 18:1/18:1, DAG 16:0/18:0, DAG 18:0/18:2, and DAG 18:0/20:4) were quantified using external calibration curves with commercially available standards, whereas the rest of the analytes were semi-quantified with the corresponding internal standards, as previously detailed [11].

### 2.5. Histological Studies

Rehydrated liver sections of 3 μm thickness, obtained from paraffin embedded samples, were stained with hematoxylin and eosin to assess the degree of necrosis. Lipid accumulation was analyzed in direct snap frozen liver sections of 6 μm thickness, stained with Oil Red O (ORO). Images were acquired with a Leica DMSL microscope equipped with a DP72 camera (Leica Microsistemas, Barcelona, Spain) and analyzed using an Image-J 1.49 device (National Institutes of Health, Bethesda, MD, USA). The area of positive ORO staining was calculated as the positively stained region per total area. All procedures were carried out at the Biobanc—Banc de Tumors i Teixits HCB IDIBAPS (Barcelona, Spain).

### 2.6. Fatty Acid β-Oxidation

Total fatty acid β-oxidation was measured as described by Lazarow [12], using 30 µg of postnuclear supernatant from the liver samples.

### 2.7. RNA Preparation and Analysis

Total RNA was isolated from different tissues using the TRIsure reagent (Bioline, Meridian Biosciences, Cincinnati, OH, USA), according to the manufacturer’s instructions. The RNA concentration was determined, and real-time polymerase chain reaction (RT-qPCR) was performed as described previously [13]. β-actin was used as the housekeeping gene to normalize the results. The primer sequences, GenBank TM numbers, and PCR product lengths are listed in the Supplementary Material (Table S2).

### 2.8. Western Blot Analysis

Protein was extracted from the tissue samples, and Western blot analysis was performed as described previously [13]. To confirm the uniformity of protein loading, the blots were incubated with an anti-vinculin antibody (Santa Cruz Biotech, Dallas, TX, USA) or an anti-β-tubulin antibody (Sigma–Aldrich, St. Louis, MO, USA) as a control for total protein extracts. A list of the antibodies used in Western blot analysis is shown in the Supplementary Material (Table S3).

### 2.9. Statistical Analysis

The sample size was determined using G*Power software (Version 3.1.9.7). The results are expressed as the mean ± standard deviation (SD). Statistical analysis was performed using GraphPad (version 8, San Diego, CA, USA). Statistical outliers were identified by Grubbs’ test (α = 0.5). Significant differences were established with an unpaired t-test. When the SD of the data was different, according to the F test, the data were transformed into their logarithms, and an unpaired t-test was rerun, or the non-parametric Mann–Whitney test was applied. Associations between study variables were tested using the Spearman correlation test. The level of statistical significance was set at *p* < 0.05.

## 3. Results

### 3.1. The Administration of the HFHFr Diet Increased Adipose Tissue Weight in Male Rats

**Figure 1:**
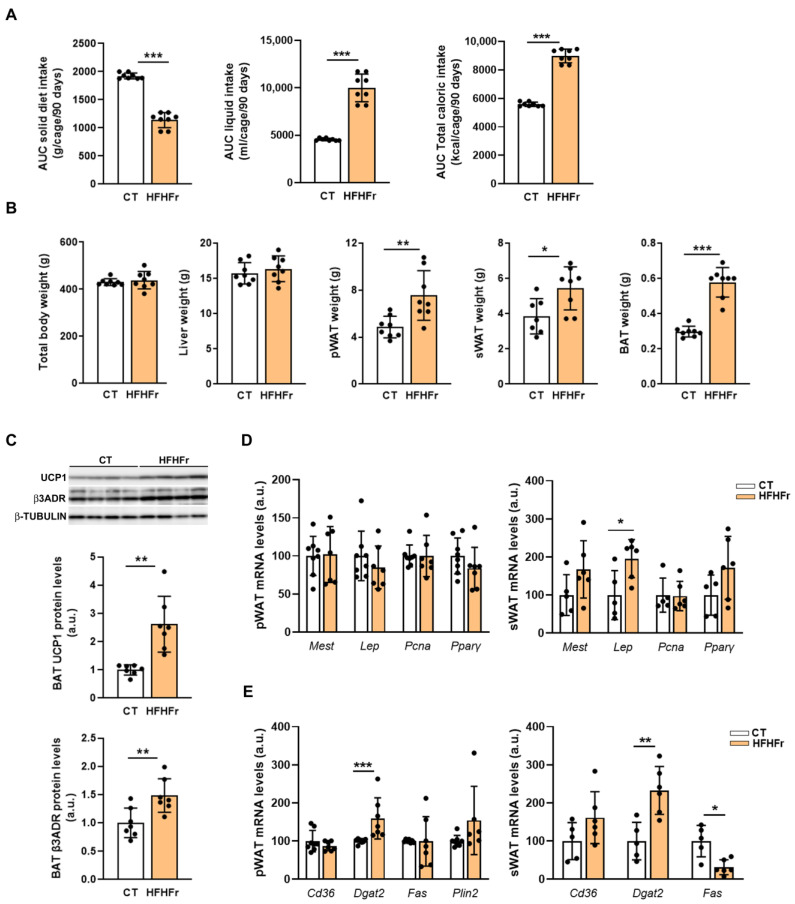
The HFHFr diet increases total caloric intake and adipose tissue weight in male rats. (**A**) Bar plots showing the area under the curve of caloric intake for the full length of the study (3 months) in control and high-fat high-fructose fed rats (*n* = 8 per group). (**B**) Bar plots showing body weight, subcutaneous and perigonadal adipose tissue weights and liver weight at the end of the experimental period corresponding to control and high-fat high-fructose fed rats (*n* = 8 per group). (**C**) Bar plots showing the content of UCP1 and β3 adrenergic receptor proteins in the brown adipose tissue obtained from control and high-fat high-fructose fed rats (*n* = 7 per group); in the upper part of the figure, representative Western blot bands corresponding to the two study groups are shown. (**D**) Bar plots showing the relative mRNA levels of *Mest*, *Lep*, *Pcna*, and *PPARγ* genes of perigonadal and subcutaneous white adipose tissue samples from control and high-fat high-fructose fed rats (*n* = 7–8 per group). (**E**) Bar plots showing the relative mRNA levels of *CD36*, *Dgat2, Fas* and *Plin2* genes of perigonadal and subcutaneous white adipose tissue samples from control and high-fat high-fructose fed rats (*n* = 7–8 per group). Data are presented as the mean ± SD. *** *p* < 0.001, ** *p* < 0.01, * *p* < 0.05 vs. control. a.u, arbitrary units; AUC, area under the curve; β3ADR, β3 adrenergic receptor; BAT, brown adipose tissue; CD36, cluster of differentiation 36; CT, control; DGAT2, diacylglycerol acyltransferase 2; FAS, fatty acid synthase; HFHFr, high-fat high-fructose; Lep, leptin; Mest, mesoderm specific transcript; Pcna, proliferating cell nuclear antigen; Plin2, perilipin2; PPARγ, peroxisome proliferator activated receptor γ; pWAT, perigonadal white adipose tissue; sWAT, subcutaneous white adipose tissue.

As in our previous study in female rats [9], male rats fed the HFHFr diet showed a reduction in solid food ingestion (×0.6), which did not compensate for the increase in the intake of liquid fructose (×2.2), leading to an increased total calorie consumption (×1.6) (Figure 1A). Despite this, the total body weight and liver weight were not significantly altered (Figure 1B). In contrast, the weights of the different WAT depots (sWAT and pWAT) were significantly increased in the rats fed the HFHFr diet (×1.4 and ×1.6, respectively). Moreover, the BAT weight was doubled in the HFHFr group compared to that of the control group (Figure 1B), with the protein expression of uncoupling protein 1 (UCP1) and β3 adrenergic receptor increased (×2.6 and ×1.5, respectively) in this tissue (Figure 1C). The increase in adiposity caused by the HFHFr diet prompted us to analyze the expression of the genes associated with adipose tissue expansion, such as leptin (Lep) and mesoderm-specific transcript (Mest), as well as the mRNA of proliferating cell nuclear antigen (Pcna) and peroxisome proliferator-activated receptor γ (PPARγ), indicative of cell proliferation and adipogenesis. As shown in Figure 1D, none of these markers were significantly altered by the diet in the WAT samples, except for the Lep mRNA levels, which were significantly increased in the sWAT. In addition, we determined the mRNA levels of the genes associated with lipid droplet formation, lipid synthesis, or lipid uptake. The results showed that in rats fed the HFHFr diet, the expression of diacylglycerol acyltransferase 2 (Dgat2) was significantly increased in both the pWAT and sWAT samples, whereas fatty acid synthase (Fas) expression was reduced only in sWAT (Figure 1E).

**Figure 2:**
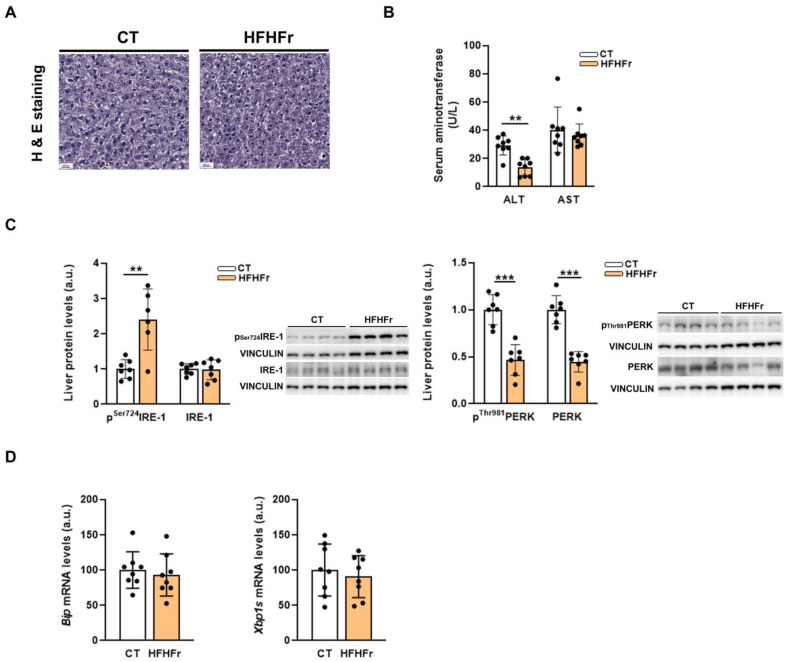
The HFHFr diet does not induce inflammation or endoplasmic reticulum stress in the liver of male rats. (**A**) Hematoxylin-Eosin (10×) representative stained liver samples corresponding to control and high-fat high-fructose fed rats. (**B**) Bar plots showing serum levels of ALT and AST of control and high-fat high-fructose fed rats (*n* = 8 per group). (**C**) Bar plots showing the content of phospho^Ser^724 and total IRE1, and phospho^Thr^981 and total PERK proteins in the liver tissue obtained from control and high-fat high-fructose fed rats (*n* = 7 per group); in the right part of the figure, representative Western blot bands corresponding to the two study groups are shown. (**D**) Bar plots showing the relative mRNA levels of *BiP* and *XBP-1s* genes of liver samples from control and high-fat high-fructose fed rats (*n* = 8 per group). Each bar represents the mean ± SD. *** *p* < 0.001, ** *p* < 0.01. ALT, alanine aminotransferase; AST, aspartate aminotransferase; a.u, arbitrary units; Bip, immunoglobulin heavy chain binding protein; CT, control; HFHFr, high-fat high-fructose; IRE1, inositol-requiring enzyme-1; PERK, protein kinase RNA-like endoplasmic reticulum kinase; XBP1s, spliced X-box binding protein 1.

No signs of hepatic damage were found in the rats fed the HFHFr diet, as observed in the liver samples stained with hematoxylin and eosin (Figure 2A). Consistently, serum aspartate aminotransferase (AST) levels were not affected, while alanine aminotransferase (ALT) serum concentration was significantly reduced (×0.5) in the HFHFr group (Figure 2B). Moreover, endoplasmic reticulum (ER) stress was not fully developed (Figure 2C): despite the hepatic protein levels of Ser^724^phosphorylated IRE1 were increased (×2.9) in the HFHFr samples, the phosphorylated and total protein kinase RNA-like endoplasmic reticulum kinase (PERK) levels were significantly reduced, and the mRNA levels of the ER stress markers immunoglobulin heavy chain binding protein (*Bip*) and spliced X-box binding protein 1 (*XBP1s*) were not modified, as previously described in females [9].

### 3.2. The HFHFr Diet Induced Hypertriglyceridemia, but Did Not Cause Hepatic Steatosis in Male Rats

**Figure 3:**
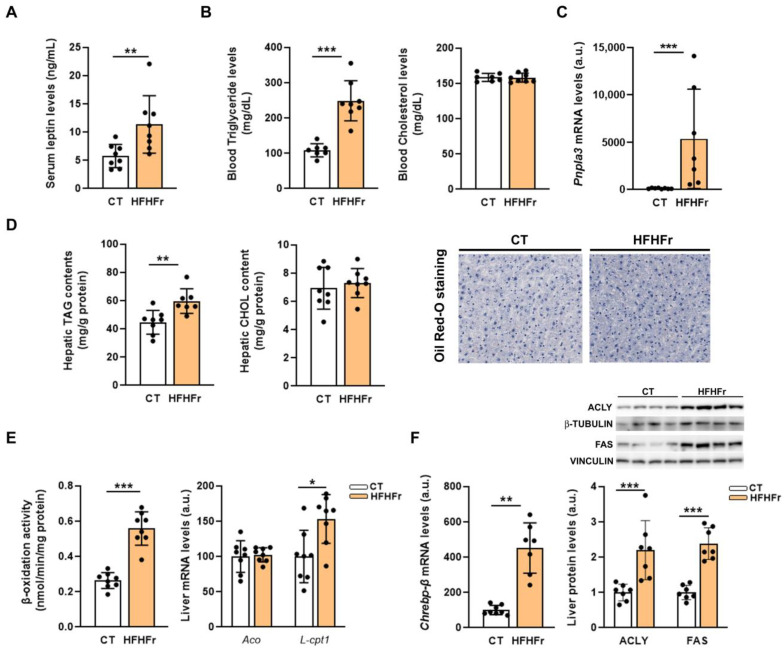
The administration of the HFHFr diet causes hyperleptinemia and hypertriglyceridemia, but not hepatic steatosis, in male rats. (**A**) Bar plot showing serum leptin levels of control and high-fat high-fructose fed rats (*n* = 8 per group). (**B**) Bar plot showing blood levels of triglycerides and cholesterol in the control and high-fat high-fructose fed rats (*n* = 7–8 per group). (**C**) Bar plots showing the relative mRNA levels of the *Pnpla3* gene of liver samples from control and high-fat high-fructose fed rats (*n* = 7–8 per group). (**D**) Bar plots showing the content of triglycerides and cholesterol as mg/g of liver protein and Oil-Red O staining of liver samples from control and high-fat high-fructose fed rats (*n* = 7–8 per group). Oil-Red O representative stained liver samples for each experimental condition are shown in the right section of the figure. (**E**) Bar plots showing fatty acid β-oxidation activity and the relative mRNA levels of *Aco* and the *L*-*Cpt1* genes of liver samples from control and high-fat high-fructose fed rats (*n* = 8 per group). (**F**) Bar plots showing the relative mRNA levels of *Chrebpβ* and protein levels of ACLY and FAS of liver samples from control and high-fat high-fructose fed rats (*n* = 7–8 per group); in the upper part of the figure, representative Western blot bands corresponding to the two study groups are shown. Each bar represents the mean ± SD. *** *p* < 0.001, ** *p* < 0.01, * *p* < 0.05 vs. control group. ACLY, ATP citrate lyase; Aco, acyl-CoA oxidase; a.u, arbitrary units; CHOL, cholesterol; Chrebp, carbohydrate response element binding protein; CT, control; FAS, fatty acid synthase; HFHFr, high-fat high-fructose; L-Cpt1, liver carnitine palmitoyl transferase I; ORO, Oil-Red O; Pnpla3, patatin-like phospholipase domain-containing 3; TAG, triglycerides.

In line with the increase in the adipose tissue mass, hyperleptinemia was observed in male rats fed the HFHFr diet (Figure 3A), whereas the leptin levels remained unaffected in female rats [9]. Leptin has been shown to regulate blood TAG concentrations by promoting VLDL secretion from the liver [14]. Accordingly, the HFHFr diet induced a more robust increase in blood TAG levels in male rats (×2.3, compared to control male rats) than that previously reported in females [9], without affecting cholesterolemia (Figure 3B). In addition, patatin-like phospholipase domain-containing protein 3 (Pnpla3) mRNA levels were increased to a higher extent in males (×53 vs. male rats fed the control diet, Figure 3C) than in female rats [9].

In contrast to the clear hypertriglyceridemia elicited by the diet, the liver TAG levels were only mildly increased (×1.3), and intrahepatic cholesterol levels remained unaffected. Moreover, ORO staining of the liver sections showed an absence of hepatic steatosis in the HFHFr group (Figure 3D). Fatty acid β-oxidation activity was increased in the livers of the HFHFr group (×2.1, Figure 3E). The gene expression of liver carnitine palmitoyl transferase 1 (*L-cpt1*), which controls mitochondrial β-oxidation, was increased by 1.5-fold, while the mRNA levels of acyl-CoA oxidase (*Aco*), the rate-limiting enzyme in peroxisomal β-oxidation, remained unchanged (Figure 3E). Regarding *de novo* lipogenesis (DNL), the mRNA levels of carbohydrate response element-binding protein β (*ChREBPβ*) were increased (×5.6) in the livers of the HFHFr group (Figure 3F). Likewise, the protein expression of the lipogenic enzymes regulated by ChREBPβ, such as ATP-citrate lyase (ACLY) and fatty acid synthase (FAS), were significantly upregulated by the HFHFr diet (×2.2 and ×2.4, respectively).

**Figure 4:**
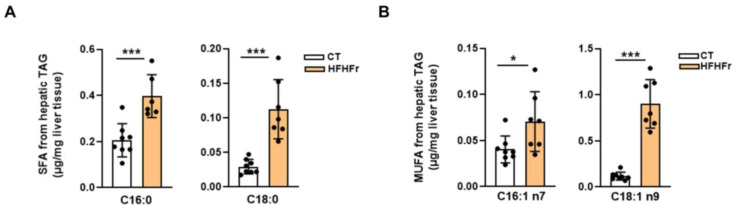
Levels of fatty acids in hepatic triglycerides from male rats. Bar plots showing the concentration of saturated (**A**) and monounsaturated (**B**) fatty acids present in liver triglycerides from control and high-fat high-fructose fed rats. Each bar represents the mean ± SD of 8 different samples per group. *** *p* < 0.001, * *p* < 0.05 vs. control group. CT, control; HFHFr, high-fat high-fructose; MUFA, monounsaturated fatty acids; SFA, saturated fatty acids.

The fatty acid composition of hepatic TAGs was significantly altered by the HFHFr diet in male rats. Thus, similar to what we observed in females [9], the diet caused an increase in the levels of several saturated fatty acids such as palmitic (×2) and stearic (×3.8) acids (Figure 4A). Moreover, monounsaturated fatty acids, such as palmitoleic and oleic acids, were also increased in the hepatic TAG fraction in the HFHFr group (×1.7 and ×7.6, respectively) (Figure 4B).

### 3.3. The HFHFr Diet Enhanced Lipolysis in the sWAT and Inhibited it in the pWAT of Male, but Not Female Rats

**Figure 5:**
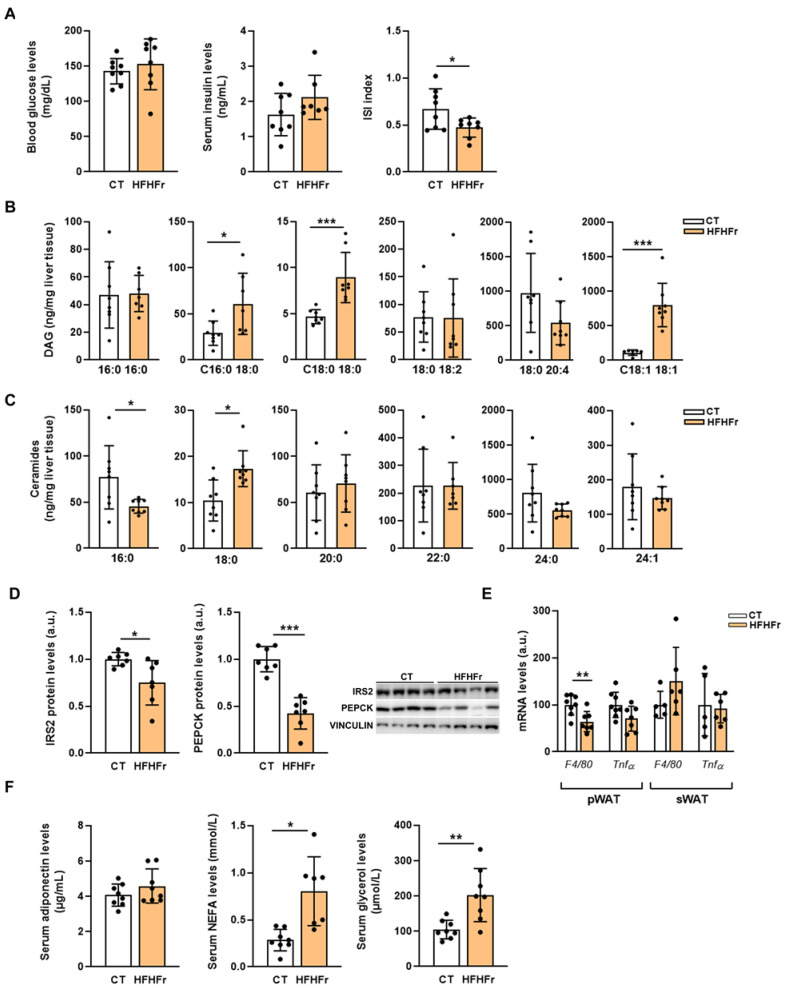
The HFHFr diet does not induce hepatic insulin resistance and adipose tissue inflammation. (**A**) Bar plots showing serum levels of insulin and blood levels of glucose, as well as the insulin sensitivity index of control and high-fat high-fructose fed rats (*n* = 8 per group). Bar plots showing the levels of (**B**) diacylglycerols and (**C**) ceramides in rat liver homogenates from control and high-fat high-fructose fed rats (*n* = 8 per group). (**D**) Bar plots showing the content of phosphorylated and total IRS2 and PEPCK proteins in the liver tissue obtained from control and high-fat high-fructose fed rats (*n* = 8 per group); in the right section of the figures, representative Western blot bands corresponding to the two different study groups are shown. (**E**) Bar plots showing the relative mRNA levels of the *F4/80* and *TNFα* genes of pWAT and sWAT samples from control and high-fat high-fructose fed rats (*n* = 6–8 per group). (**F**) Bar plots showing serum levels of adiponectin, NEFA, and glycerol from control and high-fat high-fructose fed rats (*n* = 7–8 per group). Each bar represents the mean ± SD. *** *p* < 0.001, ** *p* < 0.01, * *p* < 0.05 vs. CT. a.u, arbitrary units; Cer, ceramides; CT, control; DAG, diacylglycerols; F4/80, F4/80 macrophage marker; HFHFr, high-fat high-fructose; IRS 2, insulin receptor substrate 2; ISI, insulin sensitivity index; NEFA, non-esterified fatty acids; PEPCK, phosphoenolpyruvate carboxykinase; pWAT, perigonadal white adipose tissue; sWAT, subcutaneous white adipose tissue; TNFα, tumor necrosis factor α.

Although the blood glucose and serum insulin levels were not significantly altered, the insulin sensitivity index (ISI) was reduced in the rats fed the HFHFr diet (Figure 5A). Moreover, the HFHFr diet caused an increase in the hepatic levels of several diacylglycerol (DAG) species: DAG-C16:0/18:0 (×2.1), DAG-C18:0/18:0 (×1.9), and DAG-C18:1/18:1 (×7.2) (Figure 5B), as well as in C18:0 ceramide, but the levels of the rest of the ceramides analyzed were either unchanged or reduced (Figure 5C). In addition, the protein levels of insulin receptor substrate 2 (IRS2) were slightly, but significantly, reduced in the HFHFr liver samples (×0.75). However, the strong reduction in the protein levels of phosphoenolpyruvate carboxykinase (PEPCK, ×0.42), the rate-limiting enzyme of hepatic gluconeogenesis (Figure 5D), precluded insulin resistance in the livers of the HFHFr-fed rats, similar to our previous results for female rats [9].

**Figure 6:**
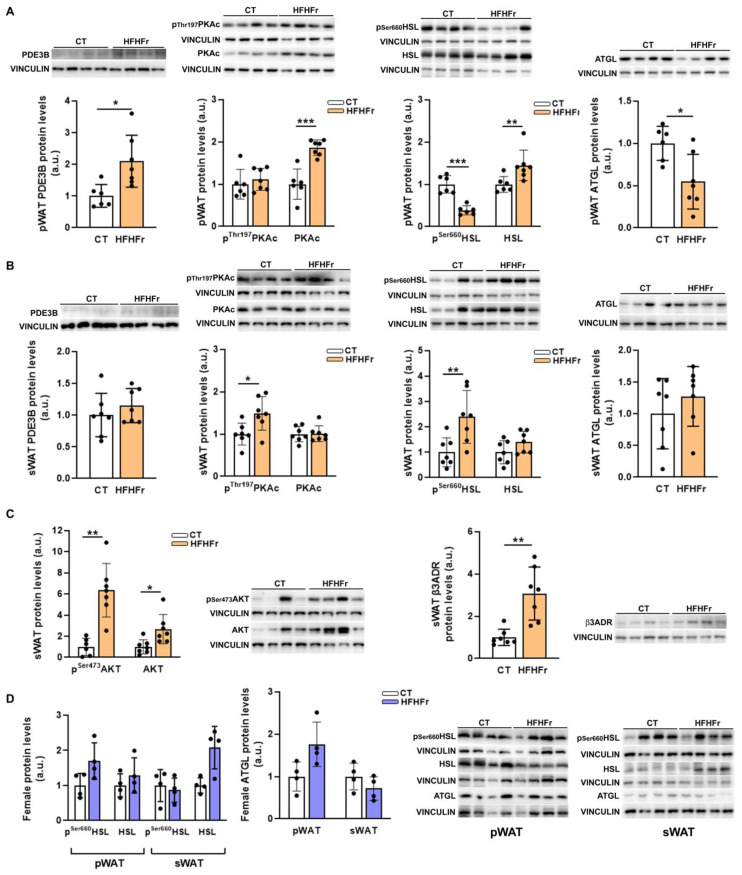
Effect of the HFHFr diet on lipolysis markers in WAT samples from male and female rats. Bar plots showing the content of PDE3B, ATGL and phosphorylated and total PKA and HSL proteins in the pWAT (**A**) and sWAT (**B**) obtained from control and high-fat high-fructose fed male rats (*n* = 6–7 per group). (**C**) Bar plots showing the content of phosphorylated and total Akt and β3ADR proteins in sWAT from control and high-fat high-fructose fed male rats (*n* = 6–7 per group). (**D**) Bar plots showing the content of ATGL and phosphorylated and total HSL proteins in the pWAT and sWAT obtained from control and high-fat high-fructose fed female rats (*n* = 4 per group). Each bar represents the mean ± SD of 6–7 different samples; in the upper-right area of the figures, representative Western blot bands corresponding to the different study groups are shown. *** *p* < 0.001, ** *p* < 0.01, * *p* < 0.05 vs. CT. a.u: arbitrary units; Akt, Akt serine/threonine kinase; ATGL, adipose triglyceride lipase; β3ADR, β3-adrenergic receptor; CT, control; HFHFr, high-fat high-fructose; HSL, hormone sensitive lipase; PDE3B, phosphodiesterase 3B; PKA, protein kinase A; pWAT, perigonadal white adipose tissue; sWAT, subcutaneous white adipose tissue.

We did not observe any signs of inflammation in any of the WAT depots, as the mRNA levels of tumor necrosis factor α (*TNFα*) were not significantly altered, and *F4/80* expression was reduced in the pWAT samples of the HFHFr group (Figure 5E). However, although the serum adiponectin concentration was unchanged, the HFHFr diet resulted in an increase in the serum levels of NEFA (×3.6) and glycerol (×1.9) (Figure 5F). Increased plasma glycerol levels indicate enhanced lipolysis, suggesting that the antilipolytic effect of insulin, which signals through the Akt/phosphodiesterase 3B (PDE3B)/protein kinase A (PKA)/hormone-sensitive lipase (HSL) pathway in the adipose tissue, was impaired in the HFHFr group. However, insulin signaling in the pWAT samples appeared to be preserved, as PDE3B expression was increased (×2), the ratio of phosphorylated to total PKA was reduced (×0.6), and consequently, there was a significant decrease in the phosphorylated levels of HSL (×0.3) (Figure 6A). Moreover, the protein levels of adipose triglyceride lipase (ATGL) were significantly reduced in the pWAT (×0.6), contributing to the suppression of lipolysis in this adipose tissue depot (Figure 6A).

The sWAT from the HFHFr-fed rats exhibited no change in PDE3B expression and an increased phosphorylation of both PKA and HSL (×1.5 and ×1.7, respectively) (Figure 6B). These results suggested impaired insulin signaling in the sWAT, but Akt phosphorylation was increased (×1.5) in this tissue (Figure 6C), precluding insulin resistance. In contrast, the stimulation of lipolysis in sWAT could be related to enhanced adrenergic signaling, as seen by the increased protein expression of the β3 adrenergic receptor (×2.4, Figure 6C). Different from the results obtained in male rats, in samples from our previous study using female rats fed the HFHFr diet [9], although there was a marginal increase in the pWAT ATGL protein levels (×1.8), the phosphorylated levels of HSL were unaltered in both adipose tissue depots (Figure 6D).

Fibroblast growth factor 21 (FGF21) is a hepatokine that regulates adipose tissue lipolysis; it has been reported that fructose-rich diets increase the circulating levels of FGF21 in rodents [15]. In our model, FGF21 serum levels were increased by the HFHFr diet in both sexes (×4.3 in females and x6 in males, Table 1). Therefore, we explored FGF21 signaling in the pWAT of both male and female rats. Early growth response 1 (*Egr1*) expression is commonly used as a readout for successful FGF21 intracellular signaling. As shown in Table 1, the mRNA levels of *Egr1* were not significantly altered in the male or female pWAT samples. Similarly, the expression of the FGF21 receptor FGF receptor 1 (*Fgfr1*) and its co-receptor β-klotho (*Klb*) was not significantly altered, although the HFHFr diet tended to increase *Klb* mRNA levels in female rats. Moreover, the expression of *Fgfr1*, *Klb,* and *Egr1* was not affected in the HFHFr sWAT samples from male rats.

## 4. Discussion

Here, we show that the three-month administration of a diet rich in plant-based fat and supplemented with 10% *w*/*v* fructose in liquid form (HFHFr diet) produced a completely different metabolic response in rats, depending on their sex. Male rats showed only a mild increase in liver TAG levels and no evidence of hepatic steatosis (as assessed by ORO staining of liver sections) when compared to rats fed a control diet. In contrast, we had previously reported that the same diet administered to female rats induced severe steatosis, as demonstrated by an 11-fold increase in liver ORO staining [9]. It is widely acknowledged that hepatic steatosis is linked to the dysregulated expression of genes controlling lipid storage and metabolism [4]. Liver lipid accretion in female rats on the HFHFr diet was attributed to increased DNL, coupled with reduced fatty acid β-oxidation [9]. In males, hepatic DNL was enhanced by the HFHFr diet to a similar extent as that in females, mainly due to the fructose-related induction of *Chrebp* and its target genes *Acly* and *Fas*. However, a striking difference was observed in fatty acid catabolism, with β-oxidation reduced in the livers of female rats fed the HFHFr diet [9], but increased in males. The increased hepatic *L-cpt1* expression in males indicated an enhanced mitochondrial β-oxidation of fatty acids. Despite no increase in *Aco* mRNA levels, the significant reduction in the concentration of long-chain polyunsaturated fatty acids in liver TAG indicated that peroxisomal β-oxidation was also increased. The levels of hepatic saturated and monounsaturated fatty acids were increased by the HFHFr diet, although the increase was quantitatively lower than that observed previously in females [9], probably due to the opposite effects resulting from the simultaneous increase in DNL and β-oxidation in males.

The crosstalk between the adipose tissue and the liver has been proposed to be a key element in the establishment of NAFLD [16]. Although the HFHFr diet similarly increased the thermogenic activity of the BAT in both male and female rats, we identified striking differences between sexes in the WAT response to this diet. Thus, our previous results in female rats did not show changes in adiposity in response to the HFHFr diet [9], whereas male rats fed the HFHFr diet exhibited significant increases in the weight of both WAT depots (perigonadal and subcutaneous) and in the expression of genes related to TAG synthesis. This difference suggests that male rats have a higher capacity to expand their WAT and store lipids in this tissue than do females, which may protect the liver from fat deposition. Expansion of adipose tissue mass in response to excessive calorie intake occurs through two mechanisms, hyperplasia (increase in cell number) and hypertrophy (increase in cell size), with the latter associated with adipocyte death and inflammation [17]. It has been shown that sex has an enormous influence on the distribution and physiology of WAT, including its expandability. Thus, the pWAT in male mice fed obesogenic diets expands by both hypertrophy and hyperplasia, whereas the sWAT expands only by hypertrophy. By contrast, the sWAT of female mice also expands by hyperplasia [18]. Despite the ponderal increase observed in our study, the markers of adipose tissue proliferation, expansion, and inflammation were not significantly altered in the sWAT or pWAT, suggesting that the HFHFr diet did not cause maladaptation or dysfunction of the WAT depots in our male rats.

**Figure 7:**
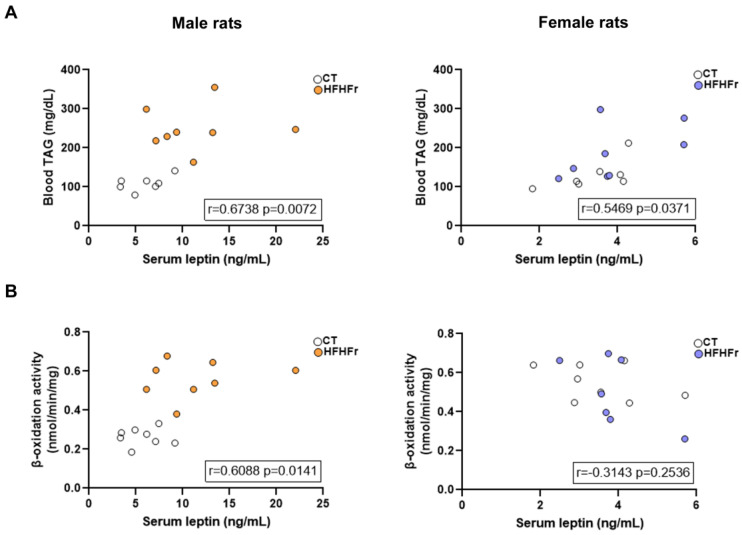
Associations between variables and serum leptin levels. Spearman correlation between (**A**) blood triglycerides and serum leptin and (**B**) β-oxidation activity and serum leptin in the control (white circles) and high-fat high-fructose (orange circles for male rats and purple circles for female rats) groups. CT, control; HFHFr, high-fat high-fructose; TAG, triglycerides.

Another sex-related difference response in our dietary model related to adipose tissue was that male rats fed the HFHFr diet exhibited higher serum leptin levels compared to control rats, which was not observed in our previous study in female rats [9]. Increased leptin levels have been linked to the stimulation of β-oxidation activity in several tissues, including the liver [19]. Moreover, a recent report shows that the injection of leptin in rats fed a high-fat diet results in reduced hepatic steatosis due to the increased export of hepatic TAG to the plasma via VLDL [14]. Interestingly, in our model, blood TAG levels were increased by the HFHFr diet in both sexes, although to a higher extent in males than in females (×2.3 and ×1.7, respectively). When we performed a correlation analysis between blood TAG and serum leptin levels, we obtained significant positive correlations in both sexes, whereas the correlation between β-oxidation activity and serum leptin was positive only in male rats (Figure 7). These results suggest that leptin production by the WAT may also be responsible for the sexual dimorphic response to the HFHFR diet. Thus, increased leptin levels in males contribute to lower hepatic lipid accumulation by simultaneously enhancing β-oxidation and TAG secretion from the liver, which in turn results in higher blood TAG levels.

The serum glycerol and NEFA levels were also higher in males, but not in females, fed the HFHFr diet [9]. It has been reported that liver PPARα may also be activated by circulating NEFA derived from adipose tissue [20]. Therefore, the higher β-oxidation activity observed in the male rats on the HFHFr diet could also be due, in part, to the activation of hepatic PPARα due to increased NEFA levels.

During fasting, the main source of plasma NEFA is the lipolysis of the TAG stored in the adipocytes [21]. The canonical lipolysis pathway consists of the consecutive hydrolysis of TAG by ATGL, HSL, and monoacylglycerol lipase (MGL), producing fatty acids and glycerol. HSL is activated by β3 adrenergic signaling, which in turn activates PKA, thereby enhancing HSL phosphorylation. However, insulin inhibits lipolysis via AKT, which stimulates PDE3B, resulting in decreased cAMP levels and the subsequent inhibition of PKA and a reduction in HSL phosphorylation [22]. When we examined lipolysis markers in the female WAT samples from our previous study, we did not find any difference between the CT and HFHFr groups, in accordance with the unaltered NEFA levels [9]. In contrast, we observed an opposite lipolytic response to the HFHFr diet in the different male WAT depots examined. In the pWAT, ATGL expression was reduced, and HSL phosphorylation was inhibited, presumably due to the insulin antilipolytic effect, resulting in an increase in PDE3B expression and reduced PKA phosphorylation. On the contrary, the sWAT exhibited increased HSL phosphorylation, indicating that this adipose tissue depot was the source of serum NEFA. However, increased lipolysis in this tissue did not seem to arise from insulin resistance, but from increased β3 signaling, as shown by the higher expression of the β3 adrenergic receptor in the sWAT from male rats on the HFHFr diet.

FGF21 serum levels were increased by the HFHFr diet in both sexes, probably because simple sugar consumption increases hepatic FGF21 synthesis by activating ChREBP [23]. One of the main targets of FGF21 is the adipose tissue, where it signals through the FGFR1receptor and its co-receptor KLB on the cell surface to initiate the intracellular signaling cascades that regulate essential functions, including lipolysis and adiponectin synthesis [24]. In our model, FGF21 signaling in the WAT of male and female rats fed the HFHFr diet did not seem to be successful, as the mRNA levels of *Egr1*, a downstream effector of the FGF21 receptor complex, were not increased in these tissues. Although the mRNA levels of *Fgfr1* and *Klb* were not significantly reduced in the WAT samples from rats fed the HFHFr diet, the lack of an Egr1 response and the fact that the adiponectin levels were not increased led us to conclude that there was resistance to FGF21 in this tissue in both sexes. In fact, it has been shown that KLB downregulation may not be the major mechanism leading to FGF21 resistance in WAT [25].

Our study has some limitations. The first is that it was conducted only in Sprague Dawley rats. Testing the effects of the HFHFr diet in other rat strains could reveal differences due to genetic background, although a previous study found that a high-fat diet induced similar qualitative metabolic changes in male Sprague Dawley and Wistar rats [26]. Our results demonstrate that sex is a key factor influencing the response to the HFHFr diet, at least in Sprague Dawley rats. The second limitation is that the effects we observed may be specific to the particular diet used. As discussed in our previous study [9], we selected a rodent diet consisting of plant-based fat, no cholesterol, and high palmitic and stearic acid content to avoid hepatic inflammation. While we can only speculate on the impacts of other high-fat diets, our goal was to characterize the effects of this specific diet on male rats and compare them to the effects observed in female rats. Focusing on this particular high-fat diet allowed us to identify clear sex differences in the development of NAFLD.

In summary, our findings highlight the influence of sex on the response to the HFHFr diet. This dietary model mimics the first phase of NAFLD (simple hepatic steatosis) in female rats, but in male rats no steatosis is evident due to the opposite effects resulting from the simultaneous stimulation of DNL and β-oxidation. Increased adiposity in males plays a key role in sexual dimorphism, with high concentrations of serum leptin and NEFA, the latter derived from increased sWAT lipolysis, contributing to enhanced hepatic fatty acid oxidation.

## Figures and Tables

**Table 1:** FGF21 levels and downstream effectors in male and female rats.

	Male Rats		Female Rats	
	CT	HFHFr	CT	HFHFr
Serum FgF21 (pg/mL)	96.3 ± 38.5	574.8 ± 1022.0 ***	154.8 ± 72.5	688.5 ± 551.0 *
*pWAT mRNA* levels (a.u)				
*Egr1*	100.0 ± 5.8	125.3 ± 93.2	100.0 ± 55.5	56.7 ± 29.4
*Fgfr1*	100.0 ± 18.3	96.7 ± 15.2	100.0 ± 10.9	112.7 ± 26.5
*Klb*	100.0 ± 37.5	69.8 ± 25.5	100.0 ± 26.6	145.3 ± 37.7
*sWAT mRNA* levels (a.u)				
*Egr1*	100.0 ± 9.48	83.1 ± 39.4	n.d	n.d
*Fgfr1*	100.0 ± 37.7	128.6 ± 61.8	n.d	n.d
*Klb*	100.0 ± 76.5	182.5 ± 117.5	n.d	n.d

The results are expressed as the mean ± SD of 8 different samples per group. * *p* < 0.05, *** *p* < 0.001; a.u, arbitrary units; CT, control; Egr1, early growth response 1; Fgfr1, FGF receptor 1; HFHFr, high-fat high-fructose; Klb, β-Klotho; n.d, not determined; pWAT, perigonadal white adipose tissue; sWAT, subcutaneous adipose tissue.

## Data Availability

The data presented in this study is contained within the article. Individual values are available from the corresponding authors upon reasonable request.

## Supplementary Materials

The following supporting information can be downloaded at: https://www.mdpi.com/article/10.3390/nu15183909/s1, Table S1: Composition of diets used in the study; Table S2: Primers used in RT-qPCR analysis; Table S3: Antibodies used in Western blot analysis.
