# Supplementary material for: Adipose Tissue Protects against Hepatic Steatosis in Male Rats Fed a High-Fat Diet plus Liquid Fructose: Sex-Related Differences
Source: Nutrients. 2023 Sep 8;15(18):3909. doi: 10.3390/nu15183909 (PMC10534325; doi:10.3390/nu15183909)
Supplement: Supplementary file 1 [file nutrients-15-03909-s001.zip › nutrients-2552688-supplementary.pdf]

Table S1. Composition of diets used in the study

Composition	2018 Teklad 18% Protein Rodent Diet	Teklad Custom Diet TD. 180456
Protein, % by weight	18.6	17.3
Carbohydrate, % by weight	44.2	41.7
Fat, % by weight	6.2	23.2
Protein, % kcal from	24	15.5
Carbohydrate, % kcal from	58	37.5
Fat, % kcal from	18	46.9
Calorie density (Kcal/g)	3.1	4.4
Cholesterol mg/kg	0	0
Carbohydrate origin	starches	starches
Fat origin	Soybean oil	Soibean oil + Cocoa butter

Table S2. Primers used in RT-qPCR analysis.

Gene	GenBank TM	Primer sequences	PCR product
**Aco**	NM_017340.2	Forward:5'-GTGAGGCGCCAGTCTGAAA-3'<br>Reverse: 5'-ACTGCTGGGTTTGAAAATCCA-3'	70 bp
**Bip**	NM_013083.2	Forward: 5'-TGATAATCAGCCCACCGTAACA-3'<br>Reverse: 5'-CAAATGTACCCAGAAGGTGATTGTC-3'	83 bp
**Egr1**	NM_012551.3	Forward:5'-GCACCTGACCACAGAGTCCTTT-3'<br>Reverse: 5'-AGAAGCGGCCAGTATAGGTGA-3'	119 bp
**Cd36**	NM_031561.2	Forward:5'-CGTATGGTGTGCTGGACATTG-3'<br>Reverse: 5'-CTTGCATGTAGGAAATGTGGAAGA-3'	79 bp
**Chrebpβ**	NM_133552.2	Forward: 5'-TGTTCAGCCGAATCTTGTCC -3'<br>Reverse: 5'-TGTTCAGCCGAATCTTGTCC -3'	125 bp
**Cpt1a**	NM_031559.2	Forward: 5'- TGCAGAACACGGCAAAATGA-3'<br>Reverse: 5'- CCGACCTGAGAGGACCTTGA-3'	70 bp
Dgat2	NM_001012345	Forward:5'-GGAACCGCAAAGGCTTTGTA-3'<br>Reverse:5'-GGAATAGGTGGGAACCAGATCA-3'	65 bp
**F4/80**	NM_001007557.1	Forward: 5'-CAGATGGGGGATGACCACAC-3'<br>Reverse: 5'-AACTCTCCGTGGTGTCAGTG-3'	116 bp
**Fas**	NM_017332.1	Forward: 5'- CCAACCGGCTCTCTTTCTTCT -3'<br>Reverse: 5'- GGCTAGAGGAGCAGGCTGTGT -3'	72 bp
**Fgfr1**	NM_024146.2	Forward:5'-GCACCTGACCACAGAGTCCTTT-3'<br>Reverse: 5'-AGAAGCGGCCAGTATAGGTGA-3'	119 bp
**Klb**	XM_039092743.1	Forward:5'- TGAGTCTGTTCTTAAGCCGGAG -3'<br>Reverse: 5'- TGCATTGGGACGGTCTTGTT-3'	140 bp
**Leptin**	NM_013076.3	Forward: 5'-GGTCACCGGTTTGGACTTCA-3'<br>Reverse: 5'-GGTCTGGTCCATCTTGGACAA-3'	67 bp
**Mest**	NM_001009617.2	Forward: 5'-GACCCTGAGGTTCCATCGAG -3'<br>Reverse:5'-ACTGATGTGGTCTCGGCTTG-3'	77 bp
**PParγ-2**	NM_001145366.1	Forward: 5'-AGCTGTGAACCACTAATATCCAAG-3'<br>Reverse:5'-GGTCCACAGAGCTGATTCCG-3'	123 bp
**PCNA**	NM_022381.3	Forward: 5'- GCAACTTGGAATCCCAGAACA -3'<br>Reverse: 5'- TCCCGGCATATACGTGCAAA -3'	79 bp

**Pnpla3**	NM_001282324.1	Forward: 5′- AGTCTCCCTCTCGATCACATCA -3′<br>Reverse: 5′- TGTTGAAGAACGGGTGGAGG -3′	94 bp
Tnfα	NM_012675.3	Forward: 5′-ATCCGAGATGTGGAACTGGC -3′<br>Reverse: 5′-CGATCACCCCGAAGTTCAGTA -3′	151 pb
**Xbp1s**	NM_001271731.1	Forward: 5′-GAGTCCGCAGCAGGTG-3′<br>Reverse: 5′-CGTCAGAATCCATGGGAA -3′	64 bp

Table S3. Antibodies used in western blot analysis.

Antibody	Reference Number
**ACLY**	Cell Signaling (# 4332)
**AKT**	Cell Signaling (# 9272)
**P(Ser473)-AKT**	Cell Signaling (# 4058)
**ATGL**	Santa Cruz (sc-365278)
**β3-AR**	Santa Cruz (sc-515763)
**FAS**	Santa Cruz (sc-55580)
**HSL**	Cell Signaling (# 4107)
**P(Ser660)-HSL**	Cell Signaling (# 4126)
**IRE1α**	Abcam (ab37073)
**p(Ser724)-IRE1α**	Abcam (ab48178)
**IRS-2**	Santa Cruz (sc-1555)
**P(Thr197)-PKA C**	Cell Signaling (#4781)
**PKAα C**	Santa Cruz (sc-28315)
**PDE3B**	Santa Cruz (sc-376823)
**PEPCK-C (I-17)**	Santa Cruz (sc-74823)
**PERK (B-5)**	Santa Cruz (sc-377400)
**P(Thr982)-PERK**	Mybiosouce (MBS9416902)
**β-TUBULIN**	Sigma-Aldrich (T4026)
**UCP1 (A-6)**	Santa Cruz (sc-518024)
**VINCULIN**	Santa Cruz (sc-73614)
